# Safety and feasibility of robotic-assisted thoracic surgery after neoadjuvant chemoimmunotherapy in non-small cell lung cancer

**DOI:** 10.3389/fonc.2023.1134713

**Published:** 2023-02-23

**Authors:** Jun Zeng, Bin Yi, Ruimin Chang, Yufan Chen, Zhongjie Yu, Yang Gao

**Affiliations:** ^1^ Department of Thoracic Surgery, Xiangya Hospital, Central South University, Changsha, Hunan, China; ^2^ Hunan Engineering Research Center for Pulmonary Nodules Precise Diagnosis and Treatment, Xiangya Hospital, Central South University, Changsha, Hunan, China; ^3^ National Clinical Research Center for Geriatric Disorders, Xiangya Hospital, Central South University, Changsha, Hunan, China; ^4^ Xiangya Lung Cancer Center, Xiangya Hospital, Central South University, Changsha, Hunan, China

**Keywords:** non-small-cell lung cancer, neoadjuvant chemoimmunotherapy, robotic-assisted thoracic surgery, video-assisted thoracic surgery, safety and feasibility

## Abstract

**Objectives:**

This study aimed to evaluate the safety and feasibility of robotic-assisted thoracic surgery (RATS) after neoadjuvant chemoimmunotherapy in NSCLC.

**Methods:**

We retrospectively collected data for NSCLC patients who received thoracic surgery after neoadjuvant chemoimmunotherapy from May 2020 to August 2022. Surgery details, pathological response, and perioperative outcome were compared between video-assisted thoracic surgery (VATS) group and RATS group. Inverse probability of treatment weighting (IPTW) was used to equal the baseline characteristics.

**Results:**

A total of 220 patients were divided into 78 VATS patients and 142 RATS patients. There was no 90-day mortality in either group. RATS patients demonstrated better results in conversion rate to thoracotomy (VATS vs. RATS: 28.2% vs. 7.5%, *P* < 0.001), number of lymph node stations harvested (5.63 ± 1.75 vs. 8.09 ± 5.73, *P* < 0.001), number of lymph nodes harvested (13.49 ± 9.325 vs. 20.35 ± 10.322, *P* < 0.001), yield pathologic-N (yp-N) assessment (yp-N0, 88.5% vs. 67.6%; yp-N1, 7.6% vs. 12.6%; yp-N2, 3.8% vs. 19.7%; *P* < 0.001), and visual analog scale pain score after surgery (4.41 ± 0.93 vs. 3.77 ± 1.21, *P*=0.002). However, there were no significant differences in pathological response evaluation for neoadjuvant chemoimmunotherapy (*P* = 0.493) and complication rate (*P* = 0.803). After IPTW-adjustment, these results remained constant.

**Conclusions:**

RATS reduced the risk of conversion to thoracotomy, provided a better yp-N stage evaluation, and improved pain score; this suggests that RATS is safe and feasible for NSCLC patients after neoadjuvant chemoimmunotherapy.

## Introduction

Non-small cell lung cancer (NSCLC) accounts for 80%–85% of all lung cancer and is one of the leading causes of cancer-related mortality worldwide (1). Approximately 22% of NSCLC patients are diagnosed with a locally advanced stage of NSCLC; the five-year survival rate of these patients is less than 33% (2). Neoadjuvant chemoimmunotherapy has been recommended as an effective treatment to improve the survival outcome of locally advanced NSCLC patients (3). In the NADIM trial, 83% of patients who received neoadjuvant chemoimmunotherapy for NSCLC achieved major pathological response (MPR), including 63% who achieved pathological complete response (pCR). The 24-month progression-free survival rate among MPR patients was 88.4%, and the overall survival rate was 100% (4). The phase III clinical trial, Checkmate816, further showed the importance of neoadjuvant chemoimmunotherapy for locally advanced NSLC patients, with a pCR rate of 24% (5).

However, neoadjuvant chemoimmunotherapy might increase the difficulty and risk of surgery. In a study by Romero et al., approximately 20% of NSCLC patients who received video-assisted thoracic surgery (VATS) as initial surgery approach ultimately converted to open thoracotomy; this figure was significantly higher than for those without neoadjuvant chemoimmunotherapy (6). In Zhang et al.’s study, 44.2% of patients who received VATS after neoadjuvant chemoimmunotherapy for NSCLC converted to thoracotomy (7). Compared with VATS, robotic-assisted thoracic surgery (RATS) has shown advantages in surgery for lung cancer, with a larger number of removed lymph nodes and more accurate N-stage assessment (8). In a previous study, the safety of RATS after neoadjuvant chemoimmunotherapy was reported to have only a 4.5% conversion rate to thoracotomy (9). However, as a single-arm study, the result was incomplete. The difference in short-term outcomes between RATS and VATS after neoadjuvant chemoimmunotherapy remains unknown. Therefore, the main objective of this study was to analyze the safety and feasibility of RATS after neoadjuvant chemoimmunotherapy in NSCLC patients.

## Materials and methods

### Study design and patient selection

This research was a retrospective study conducted at Xiangya Hospital, Central South University, and was designed to evaluate the safety and feasibility of RATS after neoadjuvant chemoimmunotherapy in NSCLC patients.

Patients who received surgery for NSCLC from May 2020 to August 2022 were included if they met the following inclusion criteria: pathological types of NSCLC were confirmed by pathology results before neoadjuvant chemoimmunotherapy; NSCLC stages before neoadjuvant chemoimmunotherapy were diagnosed as IIA–IIIB (American Joint Committee on Cancer, 8th edition) (10); received three cycles neoadjuvant chemoimmunotherapy, with PD-1/PD-L1 immune checkpoint inhibitors plus platinum-based doublet chemotherapy; and their Eastern Cooperative Oncology Group performance-status score before neoadjuvant chemoimmunotherapy was 0 or 1. Patients were excluded if they met any of the exclusion criterion as follows: aged < 18 years old; stage IIIB patients who were diagnosed with N3 lymph node metastasis positive; chose thoracotomy as the initial surgical approach; received extra medicine for neoadjuvant chemoimmunotherapy at the same time; or clinical data was incomplete.

### Therapy procedures

All patients received PD-1/PD-L1 immune checkpoint inhibitors combined with platinum-based doublet chemotherapy as neoadjuvant chemoimmunotherapy. Chemoimmunotherapy drugs were given on the first day of each treatment cycle (21 days per cycle). A standard staging evaluation was performed before and after neoadjuvant chemoimmunotherapy, including a computed tomography (CT) scan (11); 18-F-fluorodeoxyglucose positron emission tomography/CT scan; magnetic resonance imaging or CT for the brain; and a bronchoscopy examination. All patients received 18-F-fluorodeoxyglucose positron emission tomography/CT scan to assess the presence of mediastinal involvement before and after neoadjuvant chemoimmunotherapy. Surgery was planned 3–7 weeks after the first day of the last treatment cycle. If there were progressive M1 or N3 metastasis after neoadjuvant chemoimmunotherapy, patients would continue medical therapy and be excluded from this study. The type of resection for the primary tumor was determined according to standard institutional procedures, including lobectomy, bronchial or vascular sleeve lobectomy, bilobectomy, and pneumonectomy. Systematic lymphadenectomy was performed in every patient. Decisions of conversion to thoracotomy were made by surgeons during operation whenever they felt necessary. Pathological responses and yield pathologic stage (yp-stage) were determined by the Department of Pathology according to resected samples.

Patients were divided into the VATS or RATS groups according to the initial surgery approach. Surgery approach was determined by patients’ will. All surgeries were performed by surgeons with extensive experience. VATS was performed in a two-port or three-port approach liberally. RATS was performed using the Da Vinci Xi surgery system (Intuitive Surgical, Inc., Mountain View, CA, USA), using the three-arm method. Patients without viable tumor cells in resected lymph nodes and primary lung cancer were defined as pCR, while less than 10% of viable tumor cells were defined as MPR, and more than 10% were defined as an incomplete pathological response (IPR) (12).

### Clinical data collection

Patients’ demographics data, clinical variables, surgical details, and pathological details were retrospectively collected. The tumor response after completing neoadjuvant chemoimmunotherapy was evaluated by Response Evaluation Criteria in Solid Tumors (RECIST) version 1.1 (13). Pain evaluation was performed at 2 h after surgery and at discharge by a visual analog scale (VAS) (14). During hospitalization, non-steroidal anti-inflammatory drugs were used for pain relief. Recovery after surgery was evaluated at discharge according to the Activities of Daily Living scale (ADL) (15). Patients with an air leak longer than five days were defined as prolonged air leaks (PAL) (16). Surgery-related complications were defined according to the Society of Thoracic Surgeons database criteria (17).

### Statistical analysis

Categoric variables were exhibited as absolute and relative frequencies. Differences between categoric variables were evaluated by χ2 test or Fisher’s exact test. Continuous variables were presented as mean and standard deviation (SD) if normally distributed and analyzed using the Student’s t-test. Otherwise, the median was used [25%–75% interquartile range (IQR)] and analyzed with a Mann–Whitney U-test. Baseline characteristics between RATS and VATS were balanced by the inverse probability of treatment weighting (IPTW). In IPTW analysis, multivariate logistic regression was used to estimate the propensity score for each patient and regress on baseline characteristics. The inverse of the predicted probability of receiving RATS was calculated as the weight (11, 18). A covariate was considered adequate balance when the standardized mean difference (SMD) score was < 0.20. A two-tailed *P*-value of < 0.05 was considered statistically significant. All data were analyzed using R version 4.1.3 software (The R Foundation for Statistical Computing, Vienna, Austria).

## Results

### Clinical characteristics of patients

**Figure 1:**
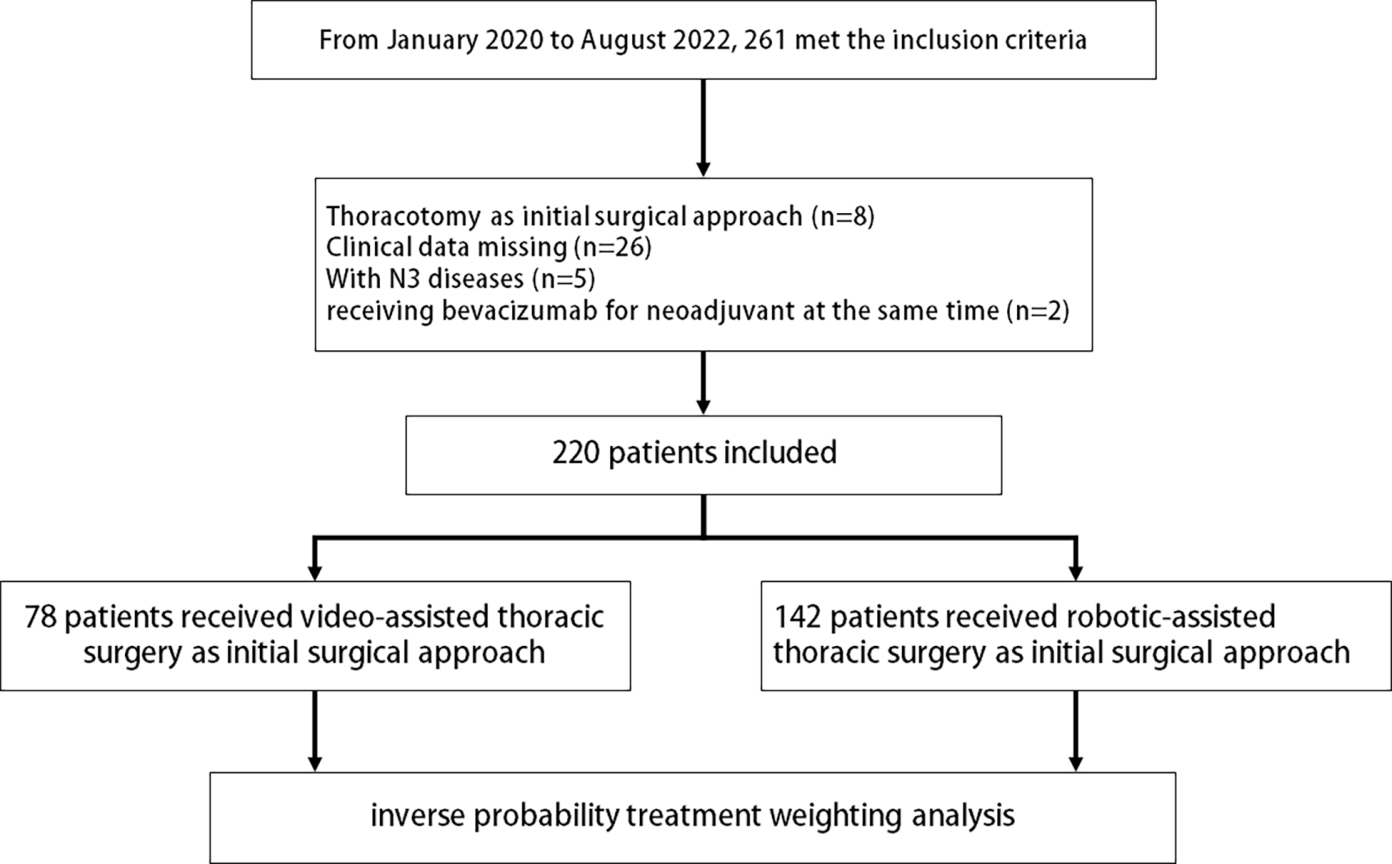
Flowchart of patient inclusion and exclusion criteria.

From May 2020 to August 2022, 261 patients were evaluated; a total of 220 patients were included in final analyses according to inclusion and exclusion criteria (**Figure 1**). Twenty-six patients were excluded due to missing data; they received neoadjuvant chemoimmunotherapy at local hospital, leading to a lack of data before neoadjuvant chemoimmunotherapy. Eight patients chose thoracotomy as the initial surgical approach. Five patients were diagnosed with positive N3 lymph node metastasis, and two received bevacizumab for neoadjuvant therapy simultaneously.

Baseline characteristics of the included patients were presented in **Table 1**. A total of 78 patients were assigned to the VATS group and 142 to the RATS group, according to the initial surgery approach. Patients were primarily male smokers, with over one-half of those in both groups being diagnosed as squamous carcinoma (SCC). Three patients had progressive disease (PD), and six patients achieved complete response (CR) before surgery, according to RECIST version 1.1. Lobectomy was the most common resection type. The two groups’ baseline characteristics were relatively balanced before IPTW. However, SMDs of some baseline characteristics were more than 0.2. IPTW was used to further equal the baseline differences between the VATS and RATS groups. After IPTW analysis, there were no baseline characteristics with SMD > 0.2 (**Table 1**).

**Table 1:** Unadjusted and IPTW adjusted patient baseline characteristics. .

Index	Without IPTW, NO. (%)				With IPTW, %			
	VATS (n=78)	RATS (n=142)	*P*	SMD	VATS	RATS	*P*	SMD
Age, mean (SD^a^), y	58.01 (8.96)	58.98 (7.56)	0.397	0.117	58.35 (8.50)	58.46 (7.66)	0.932	0.013
Gender, No. (%)			0.779	0.064			0.753	0.052
Female	15 (19.2)	31 (21.8)			23.0%	20.9%		
Male	63 (80.8)	111 (78.2)			77.0%	79.2%		
BMI, mean (SD), kg/m2	23.87 (2.88)	23.53 (3.02)	0.421	0.114	23.73 (2.65)	23.71 (3.05)	0.971	0.005
Smoking history			0.56	0.151			0.927	0.014
Never	28 (35.9)	41 (29.8)			30.6%	31.3%		
Former/current	50 (64.1)	101 (70.1)			69.4%	68.7%		
Surgery history			0.867	0.024			0.745	0.050
Never	63 (80.8)	116 (81.7)			83.1%	81.1%		
Former	15 (19.2)	26 (18.3)			16.9%	18.8%		
Tumor position			0.781	0.264			0.995	0.116
RUL	22 (28.2)	32 (22.5)			24.3%	25.0%		
RML	5 (6.4)	10 (7.0)			4.9%	5.7%		
RLL	17 (21.7)	23 (16.2)			18.5%	17.3%		
LUL	20 (25.6)	37 (26.1)			28.2%	26.4%		
LLL	2 (2.6)	1 (0.7)			0%	0.5%		
RCTC	10 (12.9)	33 (23.2)			21.2%	21.6%		
LCTC	2 (2.6)	6 (4.2)			3.0%	3.5%		
Histology			0.859	0.077			0.982	0.029
Squamous	52 (66.7)	99 (69.7)			68.1%	66.8%		
Adenocarcinoma	23 (29.5)	37 (26.1)			27.9%	29.2%		
Other^b^	3 (4.1)	6 (4.2)			4.1%	4.1%		
T stage before neoadjuvant treatment			0.979	0.062			0.959	0.085
T1	7 (9.0)	15 (10.6)			9.0%	9.8%		
T2	24 (30.8)	41 (28.9)			31.0%	29.4%		
T3	27 (34.6)	49 (34.5)			30.7%	34.0%		
T4	20 (25.6)	37 (26.1)			29.3%	26.8%		
N stage before neoadjuvant treatment			0.386	0.189			0.962	0.042
N0	8 (10.3)	8 (5.6)			6.9%	8.0%		
N1	30 (38.5)	52 (36.6)			36.3%	35.8%		
N2	40 (51.3)	82 (57.7)			56.8%	56.2%		
T stage before surgery			0.414	0.283			0.971	0.112
T0	1 (1.3)	5 (3.5)			2.3%	2.7%		
T1	25 (32.1)	56 (39.4)			35.9%	36.5%		
T2	28 (35.9)	35 (24.6)			30.2%	30.4%		
T3	12 (15.4)	21 (14.7)			12.3%	14.7%		
T4	12 (15.4)	25 (17.6)			19.3%	15.7%		
N stage before surgery			0.739	0.108			0.990	0.022
0	14 (17.9)	20 (14.1)			14.8%	15.5%		
1	26 (33.3)	48 (33.8)			32.6%	32.4%		
2	38 (48.7)	74 (52.1)			52.7%	52.0%		
RECIST evaluation			0.455	0.233			0.916	0.107
CR	1 (1.3)	5 (3.5)			2.3%	2.7%		
PR	41 (52.6)	85 (59.9)			55.2%	57.8%		
SD^c^	3 5 (44.9)	50 (35.2)			41.7%	37.9%		
PD	1 (1.3)	2 (1.4)			0.8%	1.7%		
Type of resection			0.248	0.297			0.930	0.110
Lobectomy	64 (82.1)	99 (69.7)			73.0%	74.3%		
Bilobectomy	7 (9.0)	24 (16.9)			14.4%	14.2%		
Sleeve lobectomy	2 (2.6)	6 (4.2)			5.8%	3.6%		
Pneumonectomy	5 (6.4)	13 (9.2)			6.8%	7.9%		

BMI, body mass index; RUL, right upper lobe; RML, right middle lobe; RLL, right lower lobe; LUL, left upper lobe; LLL, left lower lobe; RCTC, right central type carcinoma; LCTC, left central type carcinoma; RESIST, response evaluation criteria in solid tumors; CR, complete response; PR, partial response; PD, Progressive disease; IPTW, inverse probability treatment weight; VATS, video-assisted thoracic surgery; RATS, robotic-assisted thoracic surgery; SMD, standardized mean difference.

a SD, standard deviation;

b including: large cell carcinoma, lymphoepithelioma-like carcinoma, not otherwise specified.

c SD, Stable Disease.

### Surgery details results

A total of 22 (28.2%) patients who underwent VATS as the initial surgery approach converted to open thoracotomy. The conversion rate was higher than for RATS patients (respectively, 28.2% vs. 7.5%, *P* < 0.001). Dense adhesion and fibrosis after neoadjuvant chemoimmunotherapy and intraoperative bleeding were the most common reason for conversion. The surgical duration of VATS was shorter than RATS (respectively, 176.94 ± 74.974 min vs. 197.28 ± 70.945 min, *P* = 0.048). The bleeding volume, transfusion rate, and transfusion volume between these two groups were similar, without statistical significance. After IPTW, the difference in conversion rate remained statistically significant (VATS vs. RATS, 33.7% vs. 8.2%, *P* < 0.001). However, the surgery duration became similar (VATS vs. RATS, 190.24 ± 82.96 min vs. 196.87 ± 72.17 min, *P* = 0.625). The details were summarized in **Table 2**.

**Table 2:** Unadjusted and IPTW adjusted surgery details.

Index	Without IPTW, NO. (%)			With IPTW, %		
	VATS (78)	RATS (142)	*P*	VATS	RATS	*P*
Surgery duration, mean (SD), min	176.94 (74.97)	197.28 (70.945)	0.048	190.24 (82.96)	196.87 (72.17)	0.625
Conversion to open, NO. (%)						
Total	22 (28.2)	10 (7.0)	<0.001	33.7%	8.2%	<0.001
Primary tumor invasion	4 (5.1)	2 (1.4)		7.1%	1.6%	
Dense adhesion and fibrosis	7 (8.9)	4 (2.8)		9.3%	3.2%	
Fibrocalcified lymph nodes	3 (3.8)	1 (0.7)		5.2%	0.5%	
Bleeding	8 (10.2)	3 (2.1)		12.1%	2.9%	
Transfusion, NO. (%)	10 (12.8)	8 (5.6)	0.063	19.3%	7.5%	0.054
Bleeding volume, Median (IQR), ML	100 (50 to 200)	50 (50 to 100)	0.053	112.3 (46.7 to 198.8)	121.7 (63.1 to 218.4)	0.184
Transfusion volume Median (IQR), ML	0 (0 to 0)	0 (0 to 0)	0.078	0 (0 to 0)	0 (0 to 0)	0.072

SD, standard deviation; IQR, interquartile range; IPTW, inverse probability treatment weight; VATS, video-assisted thoracic surgery; RATS, robotic-assisted thoracic surgery.

### Pathological details and oncologic staging

The number of lymph node stations harvested was lower in VATS than RATS (respectively, 5.63 ± 1.75 vs. 8.09 ± 5.73, *P* < 0.001). Similarly, the lymph node harvested count in VATS group was lower than the RATS group (respectively, 13.49 ± 9.325 vs. 20.35 ± 10.322, *P* < 0.001). Overall yp-N staging was significantly higher in the RATS group (VATS vs. RATS; yp-N0, 88.5% vs. 67.6%; yp-N1, 7.6% vs. 12.6%; yp-N2, 3.8% vs. 19.7%; *P* < 0.001). However, there was no statistically significant difference in the yp-T staging and pathological response evaluation for neoadjuvant chemoimmunotherapy. After IPTW, these differences between these two groups were consistent, showing the stability of our results (**Table 3**).

**Table 3:** Unadjusted and IPTW adjusted pathological details and oncologic staging.

Index	Without IPTW, NO. (%)			With IPTW, %		
	VATS(n=78)	RATS(n=142)	*P*	VATS	RATS	*P*
Lymph node station count, mean (SD)	5.63 (1.75)	8.09 (5.73)	<0.001	5.64 (1.89)	7.98 (5.40)	<0.001
Lymph nodes count, mean (SD)	13.49 (9.32)	20.35 (10.32)	<0.001	13.65 (9.44)	19.92 (10.05)	<0.001
yp-T stage			0.885			0.827
yp-T0	39 (50.0)	73 (51.4)		50.6%	50.9%	
yp-T1	23 (29.5)	42 (29.6)		24.4%	28.8%	
yp-T2	12 (15.4)	20 (14.1)		19.0%	15.2%	
yp-T3	3 (3.8)	3 (2.1)		4.1%	2.0%	
yp-T4	1 (1.3)	4 (2.8)		1.9%	3.2%	
yp-N stage			<0.001			0.015
yp-N0	69 (88.5)	96 (67.6)		86.5%	65.9%	
yp-N1	6 (7.7)	18 (12.7)		7.8%	14.8%	
yp-N2	3 (3.8)	28 (19.7)		5.6%	19.3%	
Pathology response			0.493			0.449
IPR	31 (39.7)	60 (42.3)		38.1%	44.7%	
MPR	9 (11.5)	23 (16.2)		12.1%	15.9%	
PCR	38 (48.7)	59 (41.5)		49.8%	39.4%	

SD, standard deviation; yp-, yield pathological-; IPR, incomplete pathological response; MPR, major pathological response; PCR, pathological complete response; IPTW, inverse probability treatment weight; VATS, video-assisted thoracic surgery; RATS, robotic-assisted thoracic surgery.

### Perioperative outcomes

No patients died within 90 days after surgery in these two groups. The VAS score at 2 h after surgery in VATS group was higher than for RATS group (respectively, 4.41 ± 0.93 vs. 3.77 ± 1.21, *P* = 0.002). However, the VAS score at discharge was not significantly different (VATS vs. RATS, 1.27 ± 0.57 vs. 1.71 ± 0.68, *P* = 0.267). Similarly, there were no statistical differences in length of stay (LOS) after surgery, activities of daily living (ADL) score at discharge, drainage volume, and drug cost between these two groups. After IPTW-adjustment, these trends remained constant (**Table 4**).

**Table 4:** Unadjusted and IPTW adjusted perioperative outcomes.

Index	Without IPTW			With IPTW		
	VATS(n=78)	RATS (n=142)	*P*	VATS	RATS	*P*
90-day mortality	0 (0)	0 (0)	>0.990	0%	0%	>0.990
LOS after surgery, mean (SD), d	6.01 (3.00)	6.78 (4.07)	0.145	6.18 (3.11)	6.75 (4.22)	0.298
VAS score after surgery, mean (SD)	4.41 (0.93)	3.77 (1.21)	0.002	4.47 (0.97)	3.7 1(1.22)	<0.001
VAS score at discharge, mean (SD)	1.71 (0.68)	1.27 (0.57)	0.267	1.69 (0.67)	1.31 (0.56)	0.375
ADL score at discharge, mean (SD)	59.49 (11.29)	62.22 (12.27)	0.106	59.60 (11.25)	62.27 (12.22)	0.138
Drainage volume, mean (SD), ML	452.05 (399.10)	439.33 (304.18)	0.791	459.00 (300.06)	431.37 (288.50)	0.461
Drug cost, mean (SD), $	1320.70 (638.73)	1579.58 (1178.16)	0.355	1336.06 (668.59)	1571.83 (1359.65)	0.087

SD, standard deviation; LOS, length of stay; VAS, visual analogue scale; ADL, activities of daily living; IPTW, inverse probability treatment weight; VATS, video-assisted thoracic surgery; RATS, robotic-assisted thoracic surgery.

### Complications outcomes

A total of 71 cases of complications were detected, including 26 cases in VATS group and 45 in RATS group. The overall complication rate was similar in patients with different initial surgery approaches (VATS vs. RATS, 33.2% vs. 31.7%, *P* = 0.803), and no difference was detected for individual complications. Pneumonia was the most common complication in both groups (VATS vs. RATS, 16.6% vs. 14.7%, *P* = 0.194). Results were similar after IPTW-adjustment based on the baseline characteristics (**Table 5**).

**Table 5:** Unadjusted and IPTW adjusted postoperative complications.

Index	Without IPTW, NO. (%)			With IPTW, %		
	VATS(n=78)	RATS(n=142)	*P*	VATS	RATS	*P*
Total	26 (33.2)	45 (31.7)	0.803	33.8%	30.5%	0.434
Pneumonia	13 (16.7)	21 (14.8)	0.193	14.1%	9.1%	0.102
Pneumothorax	5 (6.4)	8 (5.6)	0.774	5.4%	5.2%	0.981
Prolonged air leak	4 (5.1)	9 (6.3)	0.169	10.6%	11.4%	0.769
Chylothorax	1 (1.3)	3 (2.1)	>0.990	1.0%	1.7%	0.417
Return to the OR	2 (2.6)	0 (0)	0.124	1.9%	0%	0.154
Pulmonary embolism	1 (1.3)	2 (1.4)	>0.990	0.9%	0.6%	0.927
Deep vein thrombosis	0 (0)	2 (1.4)	0.540	0	1.3%	0.162

OR, Operation room; IPTW, inverse probability treatment weight; VATS, video-assisted thoracic surgery; RATS, robotic-assisted thoracic surgery.

## Discussion

This study compared the safety and feasibility of RATS and VATS as initial surgery approaches for NSCLC after neoadjuvant chemoimmunotherapy. The results depict that the conversion rate of VATS was significantly higher than that of RATS. Moreover, the numbers of lymph node stations harvested and lymph nodes harvested in RATS were significantly higher than VATS, leading to an overall yp-N upstaging. Furthermore, the VAS pain score at 2 h after surgery was lower in RATS. Through IPTW, we further balanced the baseline characteristics. These results remained consistent, indicating the stability of our results.

In recent years, neoadjuvant chemoimmunotherapy has dramatically changed the treatment for locally advanced NSCLC, with an extraordinary pathological response rate and survival improvement (19). However, increasing numbers of studies have demonstrated that neoadjuvant chemoimmunotherapy could cause vascular fragility, inflammatory changes in hilar structures, loss of planes, and adhesions, which increased the difficulty and risk of surgery (20). Although VATS after neoadjuvant chemoimmunotherapy was considered a safe and feasible approach, there were obvious disadvantages in the high conversion rate to thoracotomy (21). In the TOP1201 clinical trial, 25% of VATS after neoadjuvant chemoimmunotherapy converted to thoracotomy (22). In the NEOSTAR clinical trail, 40% of surgeries after neoadjuvant immunotherapy for NSCLC were considered more difficult (23). In the most recent trial, the conversion rate of VATS after neoadjuvant immunotherapy for NSCLC was 11% (5). Compared with traditional VATS equipment, the Da Vinci robotic-assisted system was designed for more complicated conditions, with a more flexible surgery system and multifaceted vision technologies (24). The flexibility and stability of this system allowed the surgeon to perform minimal surgery more smoothly, particularly in complicated operations. The study by Qiu et al. revealed the safety and feasibility of robotic-assisted sleeve lobectomy, which was considered to be the most complicated type of resection in NSCLC patients (25). Similarly, our results indicated that RATS reduced the conversion risk to thoracotomy in surgery after neoadjuvant chemoimmunotherapy for NSCLC.

In addition to the conversion rate, concerns about lymph node assessment have traditionally been a drawback for VATS in NSCLC, which was an important part of the surgical treatment. According to the guidelines for NSCLC surgery, at least three mediastinal stations and three hilar stations of lymph nodes should be harvested (26). The study by Liang et al. demonstrated that a higher number of lymph nodes harvested could improve lymph node assessment and improve the survival of stage I–III NSCLC (27). RATS was considered advantageous for lymph node assessment in NSCLC without neoadjuvant chemotherapy. In Shahin et al.’s study, RATS provided a better N2 lymph nodes metastasis assessment in I–II NSCLC patients (28). Veronesi et al. further compared the difference between RATS and VATS in lymph node assessment; RATS was found to associate with a higher number of removed lymph node stations, hilar lymph nodes, and mediastinal lymph nodes (29).

Our study demonstrated more numerous removed lymph nodes in RATS after neoadjuvant chemoimmunotherapy, which led to an overall yp-N upstaging. This might be because the surgeon could easily identify lymph nodes and resect them more completely in RATS. Although there was no significant difference in pathological response evaluation between these two groups, patients might still benefit from yp-N upstaging. For example, treatment after surgery for patients with residual cancer cells positive in lymph nodes but no residual cancer cells in the primary tumor was determined according to the yp-N stage alone. This was rare in the era of neoadjuvant chemotherapy, but pure residual cancer cells positive lymph nodes would become increasingly common with the clinical application of neoadjuvant chemoimmunotherapy. Thus, lymph node upstaging might ultimately affect the survival of NSCLC patients who have received neoadjuvant chemoimmunotherapy.

Complications have been a common problem in neoadjuvant therapy. In 2015, Yang et al. reported the surgery outcomes of 84 NSCLC patients who received neoadjuvant chemotherapy; the overall complication rate was 17.9% (30). A meta-analysis further confirmed the risk of complication after surgery in NSCLC patients who received neoadjuvant chemotherapy (31). In this study, we compared the perioperative outcome of VATS and RATS. The complication rate was similar in these two groups, which suggested that the surgery approach might not be the solution to the complication rate in NSCLC patients who received neoadjuvant chemoimmunotherapy.

In addition, several issues raised in this study should be noted. First, evaluation before surgery by CT imaging might underestimate the efficacy of neoadjuvant chemoimmunotherapy. Among all the included patients, only 6 out of 220 people achieved CR, according to RESIST 1.1. However, through pathology detection after surgery, there were 97 patients who reached pCR. Second, although no statistically significant differences were found for transfusion, amount of bleeding during surgery, and blood transfusion volume between the VATS and RATS groups, the latter patients might still benefit to some degree. Third, the surgery duration was longer in RATS. However, after IPTW, the two groups’ results became similar, indicating that surgery duration might be associated with baseline characteristics.

Furthermore, this study was limited by its retrospective nature. On the one hand, potential biases remained in this study, although the baseline characteristics of the two groups were balanced by IPTW. On the other hand, we collected data for the lymph node count number from the pathological reports; this might be underestimated due to the difficulty of isolating them from lung tissue or overestimated as a result of nodal tissue fragmentation. In addition, the data for complications were prospectively extracted from the patient charts, some minor complications might have been unrecorded, such as cough or arrhythmia. Moreover, the surgical treatment of IIA–IIIB NSCLC patients after neoadjuvant chemoimmunotherapy remains probably open surgery considering the challenging of this type of surgery. However, minimally invasive surgery has become the first choice for NSCLC patients, with lower complication rate and shorter length of stay after surgery compared with open surgery, after several decades of development in modern medical technology.

The results of this retrospective study reveal that RATS was safe and feasible for IIA–IIIB NSCLC patients after neoadjuvant chemoimmunotherapy. RATS was found to have a lower conversion rate to thoracotomy, a higher count of lymph node stations and lymph nodes harvested, more-accurate yp-N staging, and a lower VAS pain score after surgery.

## Data availability statement

The raw data supporting the conclusions of this article will be made available by the authors, without undue reservation.

## Ethics statement

The studies involving human participants were reviewed and approved by the Institutional Review Board and Ethics Committee of Xiangya Hospital, Central South University, China (202210229). The patients/participants provided their written informed consent to participate in this study.

## Author contributions

JZ: Conceptualization; Data collection; Formal analysis; Writing original draft; Writing—review and editing. BY: Conceptualization; Data collection; Formal analysis; Writing original draft; Writing—review and editing. RC: Data collection; Formal analysis; Writing—review and editing. YC: Data collection; Writing—review and editing. ZY: Data collection; Writing—review and editing. YG: Conceptualization; Writing—review and editing. All authors contributed to the article and approved the submitted version.
