# Erratum: Safety and feasibility of robotic-assisted thoracic surgery after neoadjuvant chemoimmunotherapy in non-small cell lung cancer

**DOI:** 10.3389/fonc.2023.1208634

**Published:** 2023-05-03

**Authors:** Frontiers Production Office

**Affiliations:** Frontiers Media SA, Lausanne, Switzerland

**Keywords:** non-small-cell lung cancer, neoadjuvant chemoimmunotherapy, robotic-assisted thoracic surgery, video-assisted thoracic surgery, safety and feasibility

Due to a production error, there was a mistake in **Table 4** as published. The heading “Index” was incorrectly repeated and the subheadings “VATS(n=78)”, “RATS (n=142)”, “P”, “VATS”, “RATS”, and “P” were positioned above the incorrect columns. The corrected **Table 4** appears below.

**Table 4:** Unadjusted and IPTW adjusted perioperative outcomes.

	Without IPTW			With IPTW		
Index	VATS(n=78)	RATS (n=142)	*P*	VATS	RATS	*P*
90-day mortality	0(0)	0(0)	>0.990	0%	0%	>0.990
LOS after surgery, mean (SD), d	6.01(3.00)	6.78(4.07)	0.145	6.18(3.11)	6.75(4.22)	0.298
VAS score after surgery, mean (SD)	4.41(0.93)	3.77(1.21)	0.002	4.47(0.97)	3.71(1.22)	<0.001
VAS score at discharge, mean (SD)	1.71(0.68)	1.27(0.57)	0.267	1.69(0.67)	1.31 (0.56)	0.375
ADL score at discharge, mean (SD)	59.49(11.29)	62.22(12.27)	0.106	59.60(11.25)	62.27(12.22)	0.138
Drainage volume, mean (SD), ML	452.05(399.10)	439.33(304.18)	0.791	459.00(300.06)	431.37(288.50)	0.461
Drug cost, mean (SD), $	1320.70(638.73)	1579.58(1178.16)	0.355	1336.06(668.59)	1571.83(1359.65)	0.087

SD, standard deviation; LOS, length of stay; VAS, visual analogue scale; ADL, activities of daily living, IPTW, inverse probability treatment weight; VATS, video-assisted thoracic surgery; RATS, robotic-assisted thoracic surgery.

The publisher apologizes for this mistake. The original version of this article has been updated.

